# Supplementary material for: Diagnoses and critical care outcomes in a rural Tanzanian high dependency unit: A prospective cohort study
Source: PLoS One. 2025 Jun 18;20(6):e0324640. doi: 10.1371/journal.pone.0324640 (PMC12176112; doi:10.1371/journal.pone.0324640)
Supplement: S1 Table — (DOCX) [file pone.0324640.s001.docx]

**S1 Table. Association of clinical scores with In-hospital mortality among patients in the high-dependency unit.**

| **Clinical scores** | **Categories** | **N (%)** | **OR (95% CI)^a^** | **P value ^a^** |
| --- | --- | --- | --- | --- |
| Universal Vital Assessment (UVA) | Low risk (0-1)  Medium risk (2-4)  High risk (5+) | 109 (22)  176 (36)  205 (42) | Reference  2.50 (1.35-4.66)  7.00 (3.89-2.72) | <0.001 |
| National Early Warning Score (NEWS) | Low risk (0-4)  Medium risk (5-6)  High risk (7+) | 109 (22)  83 (17)  297 (61) | Reference  2.24 (1.01-4.96)  8.25 (4.34-5.67) | <0.001 |
| Modified Early Warning Score (MEWS) | Low risk (0-4)  High risk (5+) | 171 (35)  313 (65) | Reference  5.19 (3.24-8.35) | <0.001 |
| Quick Sequential Organ Failure Assessment (qSOFA) | Low risk (0-1)  High risk (2+) | 236 (48)  254 (52) | Reference  3.37 (2.28-4.98) | <0.001 |

^a^Odds Ratio (OR) and 95% Confidence Interval (CI) obtained from logistic regression
