# Supplementary material for: Diagnoses and critical care outcomes in a rural Tanzanian high dependency unit: A prospective cohort study
Source: PLoS One. 2025 Jun 18;20(6):e0324640. doi: 10.1371/journal.pone.0324640 (PMC12176112; doi:10.1371/journal.pone.0324640)
Supplement: S2 Table — (DOCX) [file pone.0324640.s002.docx]

**S2 Table. Diagnostic tests performed on patients in the high-dependency unit.**

| **Diagnostic tests** | **Total** | **Abnormal, N (%)** |
| --- | --- | --- |
| Lung Ultrasound | 159 | 122 (76) |
| Point of care ultrasound (POCUS)^a^ | 139 | 87 (63) |
| Echocardiography | 69 | 51 (74) |
| Electrocardiogram | 46 | 35 (76) |
| Head-CT scan | 35 | 26 (74) |
| Chest X-Ray | 12 | 9 (75) |
| All tests | 460 | 330 (72) |

^a^12 had Extended Focused Assessment with Sonography in Trauma (eFAST)
