# Supplementary material for: Diagnoses and critical care outcomes in a rural Tanzanian high dependency unit: A prospective cohort study
Source: PLoS One. 2025 Jun 18;20(6):e0324640. doi: 10.1371/journal.pone.0324640 (PMC12176112; doi:10.1371/journal.pone.0324640)
Supplement: S3 Table — (DOCX) [file pone.0324640.s003.docx]

**S3 Table. Treatment and medication among patients in the high-dependency unit.**

| **Category** | **Treatment/Medication** | **N (%)** |
| --- | --- | --- |
| Treatment (N=491) | Blood transfusion | 111 (23) |
|  | Vasoactive drugs | 62 (13) |
|  | Cardiopulmonary resuscitation (CPR)^a^ | 38 (8) |
|  | Noninvasive ventilation | 25 (5) |
|  | Nebulization | 11 (2) |
|  | Dialysis | 9 (2) |
|  | Atropine | 2 (0.4) |
| Medication (N=345) | Antibiotics | 201 (58) |
|  | Painkillers | 109 (32) |
|  | Antihypertensives | 79 (23) |
|  | Heart failure drugs | 68 (20) |
|  | Furosemide | 64 (19) |
|  | ACE inhibitors | 50 (14) |
|  | Spironolactone | 30 (9) |
|  | Antiretroviral therapy | 20 (6) |
|  | Aspirin | 18 (5) |
|  | Steroids | 15 (4) |
|  | Beta blockers | 12 (3) |
|  | Anti-tuberculosis drugs | 10 (3) |
|  | Antiepileptics | 5 (1) |
|  | Phenobarbital | 5 (1) |

^a^Return of spontaneous circulation (ROSC): n=2
