# Supplementary material for: Diagnoses and critical care outcomes in a rural Tanzanian high dependency unit: A prospective cohort study
Source: PLoS One. 2025 Jun 18;20(6):e0324640. doi: 10.1371/journal.pone.0324640 (PMC12176112; doi:10.1371/journal.pone.0324640)
Supplement: Attachment — Submitted filename: A Review for Manuscript Number PONE.docx [file pone.0324640.s004.docx]

**A Review for Manuscript Number PONE-D-25-05201**

"Diagnoses and outcomes of critically ill patients admitted to a high dependency unit of a rural referral hospital in Tanzania: A prospective cohort study"

**Title**

Current Title: Diagnoses and outcomes of critically ill patients admitted to a high dependency unit of a rural referral hospital in Tanzania: A prospective cohort study

Comments:

Consider making the title more concise. For example:

"Critical Care Outcomes in a Rural Tanzanian High Dependency Unit: A Prospective Cohort Study"

Alternatively, emphasize the novelty of the study:

"First Insights into Critical Care Outcomes in a Rural Tanzanian High Dependency Unit: A Prospective Cohort Study"

**Abstract**

Comments:

Background: Briefly mention the gap in literature (e.g., lack of data on HDUs in rural sub-Saharan Africa).

Methods: Clarify the sample size earlier (e.g., “491 patients admitted to the HDU”).

Results: Highlight the most striking finding (e.g., “Mortality during HDU stay was 30%, with sepsis and stroke being the deadliest conditions”).

Conclusion: Add a sentence on implications for policy or practice (e.g., “These findings underscore the need for improved critical care resources in rural settings”).

**Introduction**

Comments:

Contextualize the Problem: Add a sentence on the burden of non-communicable diseases (NCDs) in Tanzania, as they are a major focus of the study.

Justify the Study: Emphasize why this study is novel (e.g., “This is the first study to describe outcomes in a rural HDU in sub-Saharan Africa”).

Clarify Objectives: Rephrase the objectives to be more specific (e.g., “To describe patient characteristics, diagnoses, and outcomes, and identify predictors of in-hospital mortality”).

**Methods**

Comments:

Study Design: Clarify the prospective nature of the study earlier in the section.

Data Collection: Provide more detail on how data quality was ensured (e.g., training of data collectors, use of standardized forms).

Statistical Analysis: Briefly explain why specific statistical methods were chosen (e.g., Cox regression for mortality predictors).

Ethics: Mention how oral consent was documented to address potential concerns about consent validity.

**Results**

Comments:

Clarify Key Findings: Highlight the most important results in the text (e.g., “Sepsis and stroke were associated with the highest mortality rates”).

Simplify Tables: Consider merging or simplifying tables to improve readability (e.g., combine Tables 1 and 2).

Visuals: Ensure figures (e.g., ROC curves) are clearly labeled and interpretable.

**Discussion**

Comments:

Structure: Use subheadings (e.g., “Key Findings”, “Comparison with Literature”, “Implications for Practice”) to improve flow.

Contextualize Findings: Discuss how the high mortality rates reflect systemic challenges in rural healthcare.

Policy Implications: Add a paragraph on how the findings can inform policy (e.g., “These results highlight the need for increased investment in rural critical care infrastructure”).

Limitations: Expand on the impact of lost-to-follow-up cases and diagnostic limitations.

**Conclusion**

Comments:

Call to Action: Add a sentence on next steps (e.g., “Future studies should explore interventions to reduce mortality in rural HDUs”).

Broader Impact: Mention how the study contributes to global health equity (e.g., “This study provides critical insights into improving healthcare access in low-resource settings”).

**References**

Comments:

Ensure all references are formatted consistently according to the journal’s guidelines.

Include more references from sub-Saharan Africa to strengthen the regional context.

**Overall Suggestions**

Language and Clarity:

- Simplify complex sentences for better readability.
- Avoid jargon and define acronyms (e.g., HDU, NEWS, qSOFA) at first use.

Figures and Tables:

- Ensure all figures and tables are high-quality and clearly labeled.
- Add a brief narrative summary for each table/figure in the text.

Data Availability:

- Clarify how readers can access the data (e.g., repository name, DOI).

Strengths and Limitations:

- Highlight the study’s strengths (e.g., prospective design, comprehensive data collection) more prominently.
- Discuss limitations in more depth (e.g., impact of financial constraints on patient outcomes).
